# Point-of-care Ultrasound Identification of Iliopsoas Abscess in Emergency Department: A Case Report

**DOI:** 10.5811/cpcem.2020.5.45255

**Published:** 2020-07-20

**Authors:** Nehal A. Al-Sadhan, Otto Liebmann, Kristin H. Dwyer

**Affiliations:** * King Fahad Medical City, Department of Emergency Medicine, Riyadh, Saudi Arabia; † Warren Alpert Medical School of Brown University, Department of Emergency Medicine, Division of Emergency Ultrasound, Providence, Rhode Island

**Keywords:** abdominal pain, flank pain, psoas abscess, ultrasound, bedside ultrasound, POCUS

## Abstract

**Introduction:**

The iliopsoas muscle is a rare place for an abscess to collect. While these abscesses can have high mortality, they are often misdiagnosed. The use of point-of-care ultrasound (POCUS) can aid in earlier diagnosis.

**Case Report:**

A 45-year-old male presented to the emergency department (ED) with severe lower back pain. The pain radiated to both of his legs and was associated with fever, weight loss, and malaise. The differential diagnosis for this patient was broad. A POCUS was performed at the bedside and revealed bilateral iliopsoas abscesses. This finding was then confirmed by computed tomography.

**Conclusion:**

In this case report we will discuss how to identify an iliopsoas abscess using POCUS in ED patients, and the utility of POCUS to facilitate an expedited diagnosis.

## INTRODUCTION

The iliopsoas muscle is located in the retroperitoneal space arising from the thoracic and lumbar vertebrae and it serves as the flexor muscle for the hip.^2^ It is rare for an abscess to collect in this space; however, males and younger individuals are more commonly affected.^2^ Iliopsoas abscesses are categorized as either primary (idiopathic) or secondary to direct spread from nearby structures.^3^ These abscesses are most commonly caused by hematogenous spread of *Staphylococcus aureus*. Patients with Crohn’s disease, acquired immunodeficiency syndrome, diabetes, immunosuppression, or intravenous drug abuse (IVDA) are at increased risk.^2^ The mortality associated with iliopsoas abscesses is higher for those resulting from hematogenous spread (19%) than those that are categorized as primary (<5%).^3^ Patients are frequently misdiagnosed as they present with nonspecific symptoms and the diagnosis may not even be considered in the differential.^2^,^3^ The classic triad is back pain, fever, and a limp; however, many patients don’t exhibit the complete triad. Incorporating ultrasound at the bedside may aid in a more rapid diagnosis, enabling earlier treatment and potentially a decrease in mortality.^4^

## CASE REPORT

A 45-year-old, ill-appearing male with no past medical or surgical history presented to the emergency department (ED) with a chief complaint of atraumatic low back pain that radiated down both of his legs. The pain started two weeks earlier and was associated with fevers and chills. Additional symptoms included difficulty with ambulation, generalized weakness, malaise, and 30 pound weight loss over several months. He denied any history of bowel or bladder incontinence, sensory deficits, or weakness. The patient denied IVDA or recent travel. On physical exam, he appeared pale and cachectic. He was febrile at 39.8º Celsius and his heart rate was 139 beats per minute. His other vital signs were normal. He had bilateral lower-back tenderness, but no bony midline pain. The skin over his back was normal appearing, with no redness, rash, or evidence of trauma. His neurological examination was within normal limits.

**Image 1:**
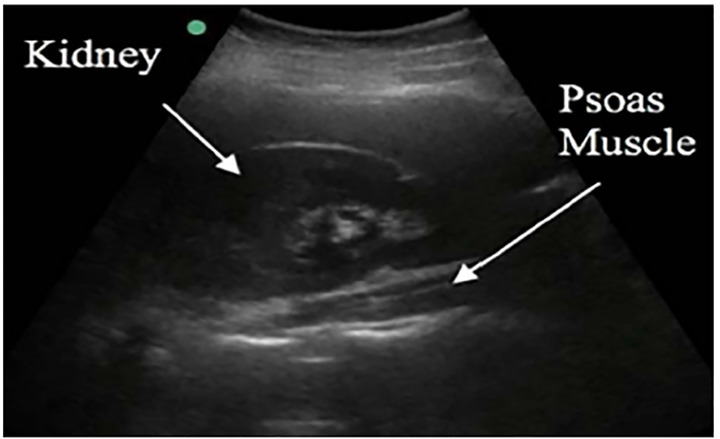
Normal right upper quadrant sonoanatomy with the psoas muscle visible posterior to the kidney.

**Image 2:**
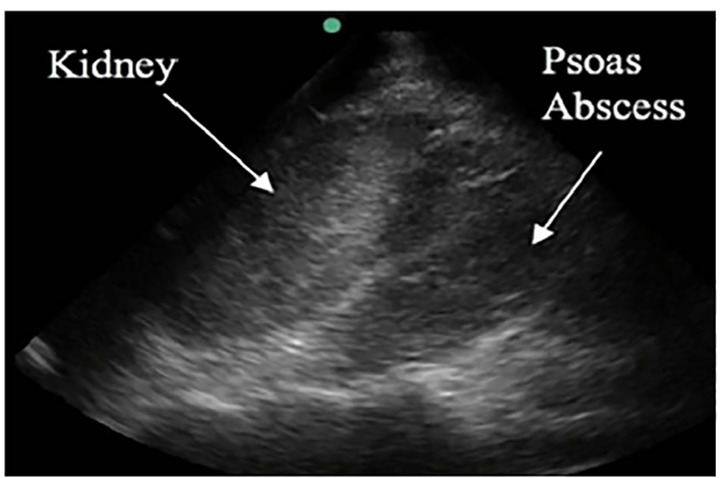
Right upper quadrant ultrasound demonstrating heterogenous mass within the psoas musculature.

**Image 3:**
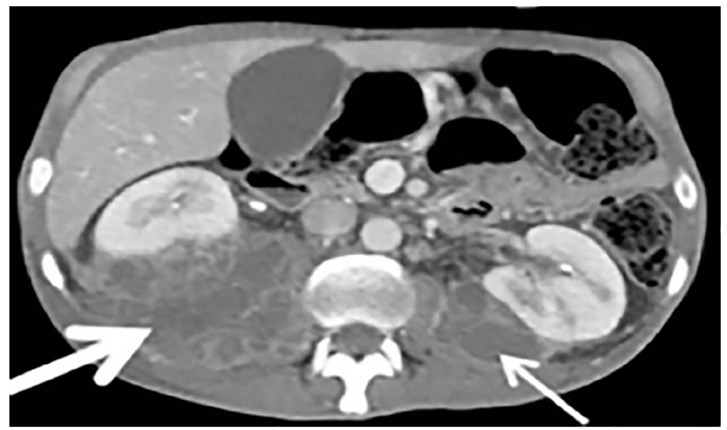
Computed tomography with intravenous contrast demonstrates bilateral psoas abscesses. The right-sided abscess is much larger as indicated by the thicker arrow.

Initial laboratory investigations revealed an elevated white blood cell count of 30,800 cells per cubic millimeter (3.5–11.00×10^9/L), with 22% bands. In addition, he had a platelet count of 714×10^9^ per liter (/L) (150–400×10^9/L), C-reactive protein of 461.36 milligrams per liter (mg/L) (RR 0–8 mg/L), and an erythrocyte sedimentation rate of 130 millimeters per hour (mm/h) (0–30mm/hr). Blood cultures sent from the ED were negative for growth at five days. Urine analysis revealed pyuria, and was positive for nitrites and leukocyte esterase, but with a negative culture. The emergency physician performed a point-of-care ultrasound (POCUS) with a curvilinear probe (2–5 megahertz) to evaluate for hydronephrosis, given the fever and back pain. While no hydronephrosis was seen, an abnormal collection of mixed echogenicity was visualized posterior to the kidney within the iliopsoas muscle (Images 1 and 2). The patient had a computed tomography (CT) that confirmed bilateral iliopsoas abscesses (Image 3) and, in addition, identified a pulmonary abscess. IV antibiotics were initiated in the ED, and the patient was admitted to the hospital.

## DISCUSSION

Iliopsoas abscesses were first described as psoitis in 1881 and were defined as “a collection of pus in the iliopsoas compartment.”^5^ The majority of clinical exam findings and laboratory tests are not specific for the iliopsoas abscess diagnosis. Importantly, mortality reaches 100% in untreated patients, making it an important condition to recognize and treat early.^1^ To assess for iliopsoas abscess on ultrasound, a curvilinear or phase array probe should be placed in the midaxillary line at the level of the xiphoid with the marker pointing to the patient’s head. Consider rotating the probe slightly oblique, parallel to the ribs, to decrease interference from rib shadow. The iliopsoas muscle will be seen as a striated structure between the kidney and the vertebral column.^6^ In this case report, a large heterogenous mass was visualized with loss of the normal muscle striation.

**CPC-EM Capsule**

What do we already know about this clinical entity?

Patients with iliopsoas abscesses are frequently misdiagnosed as they present with nonspecific symptoms. The classic triad is back pain, fever, and a limp; however, many patients don’t exhibit the complete triad.

What makes this presentation of disease reportable?

Mortality of untreated iliopsoas abscesses is very high, making it an important condition to recognize and treat early. In this case, we were able to rapidly diagnose an iliopsoas abscess utilizing point-of-care ultrasound (POCUS), and confirm by computed tomography.

What is the major learning point?

Incorporating POCUS may aid in a more rapid diagnosis of iliopsoas abscesses, enabling earlier treatment and potentially a decrease in mortality.

How might this improve emergency medicine practice?

POCUS may be able to aid in earlier identification of this dangerous condition, which could limit the delay in diagnosis and management of these patients.

It can be difficult to differentiate hematoma from abscess or other masses on ultrasound. However, if there is an abnormal collection identified on ultrasound within the psoas in the setting of fever, back pain, and limp, clinical suspicion for an abscess should be high. Currently, CT remains the definitive diagnostic and is considered the “gold standard” for diagnosis.

## CONCLUSION

The diagnosis of iliopsoas abscess is often delayed because of the non-specific presentation. For immunocompromised, febrile patients with back pain, maintain a high level of clinical suspicion. Those with Crohn’s disease are at particularly high risk. POCUS may be able to aid in earlier identification of this dangerous condition, which could limit the delay in diagnosis and management of these patients. Infected kidney stone will be on the differential diagnosis for many of these patients, so clinicians should be interrogating the kidney at the bedside already with POCUS.
